# Probability of outbreaks and cross-border dissemination of the emerging pathogen: a genomic survey of *Elizabethkingia meningoseptica*

**DOI:** 10.1128/spectrum.01602-23

**Published:** 2023-10-10

**Authors:** Shaohua Hu, Yingying Chen, Hao Xu, Jing Chen, Shaojun Hu, Xiaohua Meng, Shujun Ni, Yonghong Xiao, Beiwen Zheng

**Affiliations:** 1 State Key Laboratory for Diagnosis and Treatment of Infectious Diseases, National Clinical Research Center for Infectious Diseases, Collaborative Innovation Center for Diagnosis and Treatment of Infectious Diseases, The First Affiliated Hospital, Zhejiang University School of Medicine, Hangzhou, Zhejiang, China; 2 Department of Neurosurgery, Shaoxing People's Hospital (Shaoxing Hospital, Zhejiang University School of Medicine), Shaoxing, Zhejiang, China; 3 Data Resource Development Department, Hangzhou Matridx Biotechnology Co., Ltd., Hangzhou, Zhejiang, China; 4 Department of Pathology, Zhejiang Provincial Hospital of Chinese Medicine, Hangzhou, Zhejiang, China; 5 Department of Structure and Morphology, Jinan Microecological Biomedicine Shandong Laboratory, Jinan, Shandong, China; 6 Research Units of Infectious Diseases and Microecology, Chinese Academy of Medical Sciences, Beijing, Hebei, China; Institut National de Santé Publique du Québec, Sainte-Anne-de-Bellevue, Québec, Canada

**Keywords:** *Elizabethkingia meningoseptica*, genome sequencing, transmission distribution, phylogenetic structure, system genomics, nosocomial outbreak

## Abstract

The emerging infectious agent *Elizabethkingia meningoseptica* is associated with life-threatening infections in immunocompromised individuals. However, there are limited data on its geographic distribution, phylogenetic evolution, pathogenesis, and transmission. In this study, we comprehensively analyze and compare the genomic features, evolutionary history, emergence date, and transmission networks of global *E. meningoseptica*. Geographical distribution reveals the presence of the emerging bacteria in Asia, Europe, and North America, three continents with similar latitudes. Phylogenetic analyses show no relationship between the strain’s evolutionary history and its location, origin, or source, despite the presence of genetic diversity. Analysis of the emergence timeline suggests that America is the most likely source of *E. meningoseptica* with the common ancestor of this pathogen dating back 90 years. Putative transmission networks indicate that *E. meningoseptica* bacteria can spread within the same hospital and even across borders. Minor variations in resistance genotypes and virulence genes are observed, supporting existing evidence of inherent resistance and pathogenicity in *E. meningoseptica*. Additionally, minocycline and doxycycline demonstrate potent antimicrobial activity against this pathogen, making them promising candidates for treating *E. meningoseptica* infections. Our research highlights the potential for severe nosocomial outbreaks caused by *E. meningoseptica* with horizontal transmission occurring between countries worldwide. To prevent future outbreak infections, increased genomic surveillance of global *E. meningoseptica* populations is necessary.

**IMPORTANCE:**

*Elizabethkingia meningoseptica* is an emerging infectious agent associated with life-threatening infections in immunocompromised individuals. However, there are limited data available on the genomic features of *E. meningoseptica*. This study aims to characterize the geographical distribution, phylogenetic evolution, pathogenesis, and transmission of this bacterium. A systematic analysis of the *E. meningoseptica* genome revealed that a common ancestor of this bacterium existed 90 years ago. The evolutionary history showed no significant relationship with the sample source, origin, or region, despite the presence of genetic diversity. Whole genome sequencing data also demonstrated that *E. meningoseptica* bacteria possess inherent resistance and pathogenicity, enabling them to spread within the same hospital and even across borders. This study highlights the potential for *E. meningoseptica* to cause severe nosocomial outbreaks and horizontal transmission between countries worldwide. The available evidence is crucial for the development of evidence-based public health policies to prevent global outbreaks caused by emerging pathogens.

## INTRODUCTION

Gram-negative bacterium *Elizabethkingia meningoseptica*, which is non-motile and exhibits catalase and oxidase positivity, as well as non-glucose fermentation, is commonly found in both natural environments and hospital settings, including water, soil, plants, foodstuffs, and medical devices (1 – 3). Initially known as *Flavobacterium meningosepticum*, *E. meningoseptica* was first identified in 1959 by Elizabeth O. King (4). It was later renamed *C. meningosepticum* in 1994 when it was classified into a new genus, *Chryseobacterium* (2). However, in 2005, through analysis of the 16S rRNA gene sequence and phylogenetic tree, it was reassigned to a new genus, *Elizabethkingia* (1). While healthy individuals rarely contract *E. meningoseptica* infections or diseases, there has been an increasing number of reports linking this bacterium to life-threatening infections in immunocompromised individuals. It has been associated with severe meningitis (particularly neonatal meningitis) (5 – 11), bacteremia (12 – 17), respiratory infection (18, 19), urinary tract infection (20), sepsis (21, 22), eye infections (23 – 26), biliary tract infections (27, 28) and has emerged as a significant public health concern. Furthermore, recent studies have described nosocomial outbreaks associated with *E. meningoseptica* (5, 6, 21, 29 – 31). It is also concerning that previous research has shown a mortality rate of up to 40% with *E. meningoseptica* infections, especially in neonates (14, 15, 32). For example, among 19 mechanically ventilated patients in acute care hospitals affected by an outbreak, 8 ultimately died (5).

Treatment of *E. meningoseptica* infection is challenging due to the lack of effective treatment options and this microorganism’s reduced sensitivity to many classes of antimicrobials. The organism is generally resistant to antimicrobials that are effective against Gram-negative bacteria, such as aminoglycosides, chloramphenicol, extended-spectrum beta-lactams, carbapenems, colistin, and even vancomycin (12, 14, 15, 33 – 37). Limited information is available regarding *E. meningoseptica’s* pathogenesis, resistance mechanisms, and direct transmission routes (38). In-depth genome analyses will provide insights into the evolutionary history, transmission pathways, pathogenesis, and resistance mechanisms of *E. meningoseptica*. Whole genome analysis can offer molecular diagnostic tools, such as single nucleotide polymorphisms (SNPs), which have potential clinical utility. *E. meningoseptica* can benefit from whole genome analysis as it is a relatively understudied bacterium with few genomes available in the NCBI database. A review of the existing literature reveals that almost all publications on this microorganism are case reports, with only a few studies investigating its comparative genomics, such as phylogenetic structure and geographical distribution. Here, we have isolated over 20 clinical *E. meningoseptica* strains and sequenced their complete genomes. Various methods were utilized to analyze and compare the genome characteristics of all GenBank sequences of *E. meningoseptica* bacteria worldwide. This collection represents the largest and most geographically diverse sample to date. Our study enables an investigation into the population structure, evolutionary history, geographical distribution, transmission assessment, virulence, and resistance mechanisms of the studied group.

## RESULTS

### Study design and bacterial isolates

The workflow of this study is presented in Fig. S1. All available global genomes of *E. meningoseptica* at the time of the study were included. The bacterial collection consisted of 47 isolates from over six countries. This collection included 25 newly sequenced genomes from our previous studies (2010–2019) and 22 publicly available genomes (up to June 2020) in GenBank of NCBI (Table S1). The strains were obtained from various sources such as blood, bronchoalveolar fluid, bile, cerebrospinal fluid, sputum, tracheal exudate, urine, and environmental samples (Table S1). Our collection of *E. meningoseptica* strains was previously identified using MALDI-TOF MS (matrix-assisted laser desorption ionization time-of-flight mass spectrometry systems) (Bruker Daltonics, USA). A heatmap was constructed based on ANI (average nucleotide identity) values to confirm our collected *E. meningoseptica* strains and minimize the impact of obtained genomes. The ANI values of all representative *E. meningoseptica* species are presented in Fig. S2. The ANI values across all selected *E. meningoseptica* strains (except one at 95.18%) on the map are >97% (Fig. S2), indicating that these strains belong to the same species according to microbial taxonomy (>95% cut-off for ANI) (39).

### Gene repertoire of *E. meningoseptica* species

**Fig 1:**
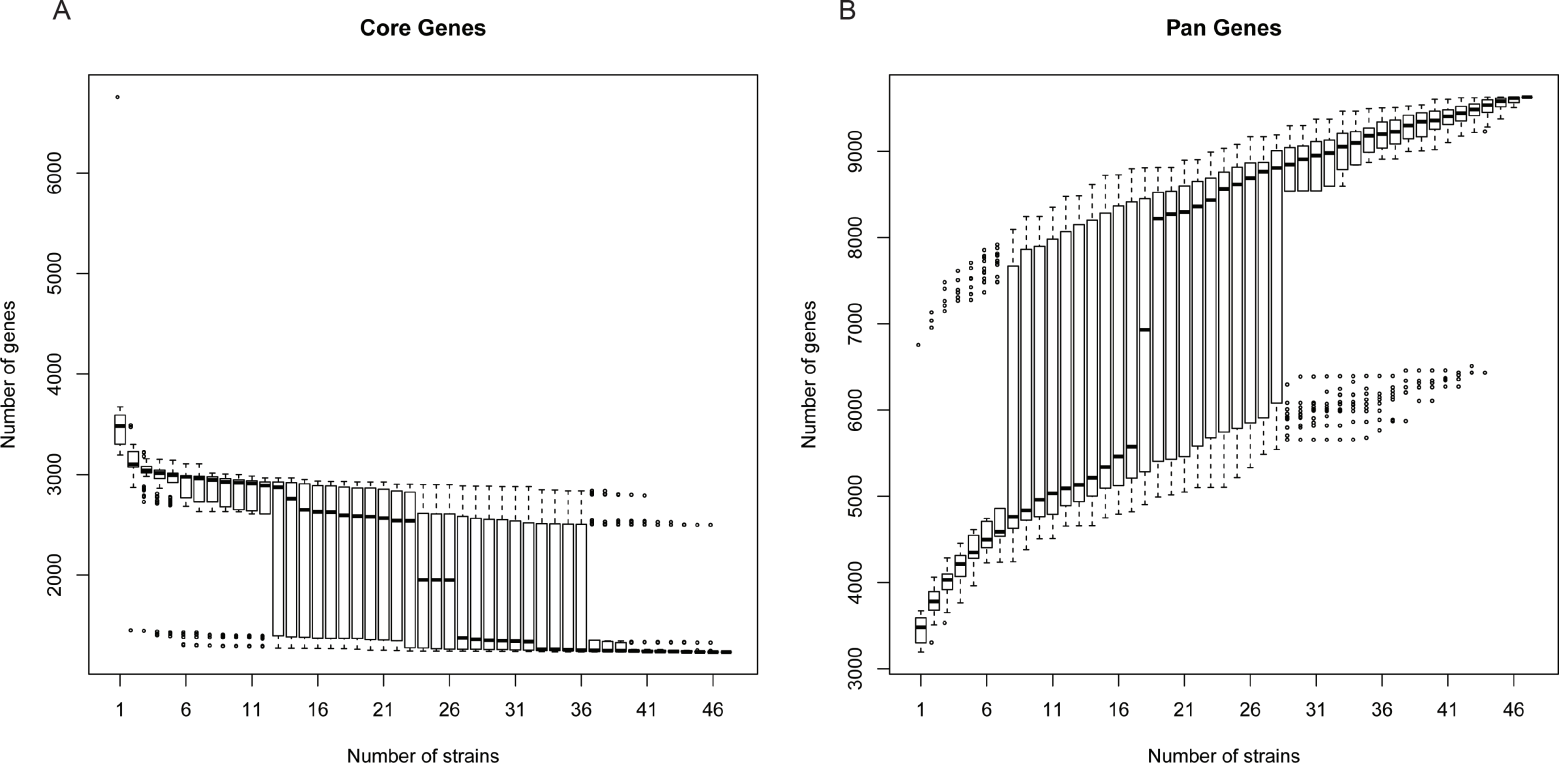
Core-genome and pan-genome evolution according to the number of *E. meningoseptica* genomes. The bar diagrams depict the changes in the total number of core-genome and pan-genome as more genomes are added. (A) The number of core-genome (shared genes) as a function of the sequentially added genomes. (B) The accumulation of the total number of genes (pan-genome) with a given number of genomes sequentially added.

The pan-genome and core-genome were sorted and used for gene repertoire analysis in all 47 global *E. meningoseptica* genomes (Fig. 1A and B). The number of shared genes (core genes) decreased as more genomes were added (see Fig. 1A). The analysis of the core-genome revealed that all *E. meningoseptica* genomes shared at least 1,233 genes. An analysis of the pan-genome showed that *E. meningoseptica* has an open pan-genome, as new genes were found when more genomes were included in the analysis (Fig. 1B). Interestingly, the distribution of specific genes among these 47 genomes was heterogeneous, ranging from 0 to 3,203. However, 17 *E. meningoseptica* isolates from different origins shared 0 specific genes, indicating that these isolates are very similar.

### Phylogenetic diversity and geographical distribution

**Fig 2:**
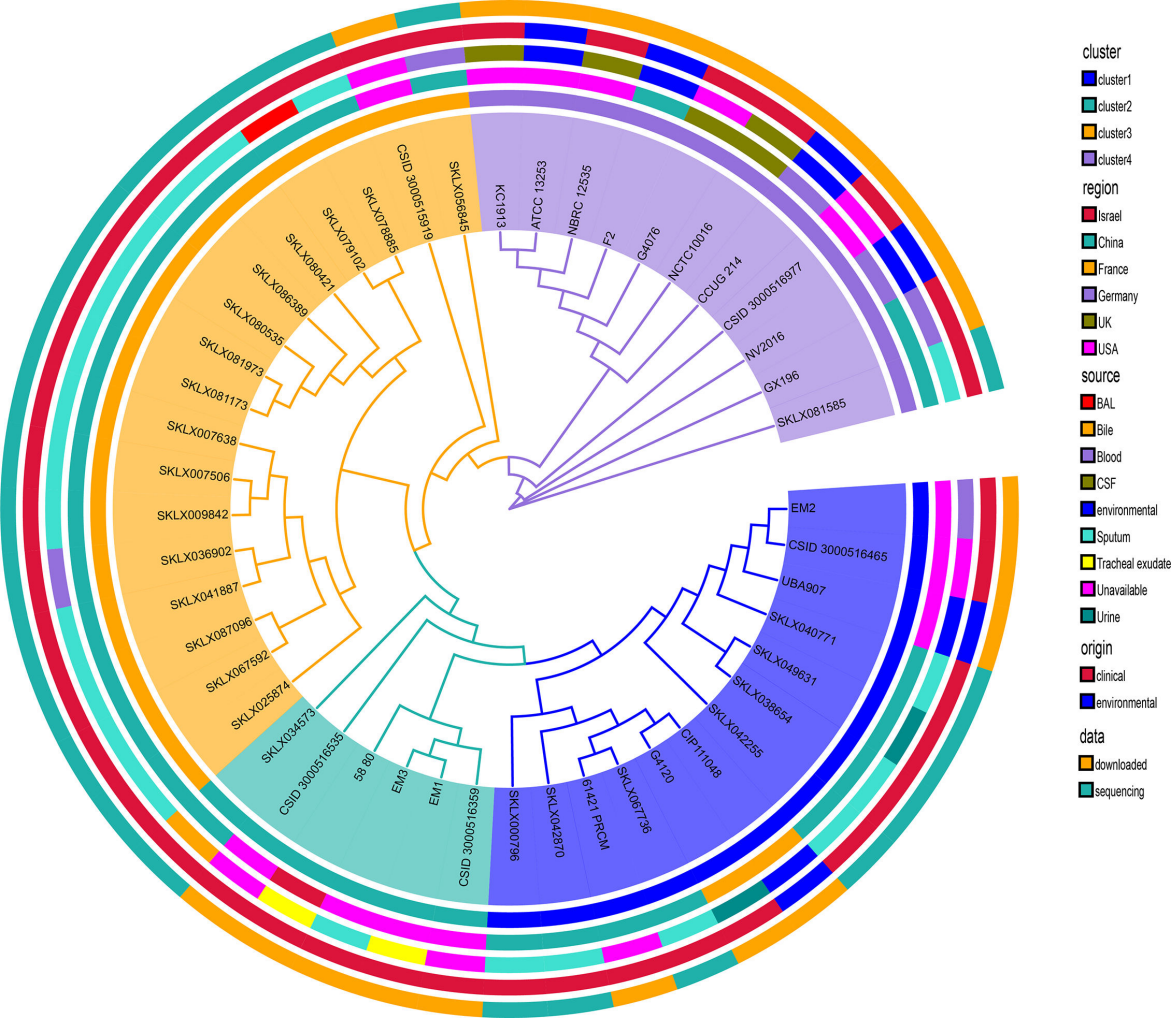
Phylogenetic structures of global *E. meningoseptica* genomes. Maximum likelihood SNP tree outlining the phylogenetic topological structure of 25 *E. meningoseptica* isolates unique to this study combined with 22 global *E. meningoseptica* isolates. Branch colors represent populations categorized into four main colors (yellow, cyan, blue, and purple). The tree is adjacent to five concentric circles highlighting associated metadata. The inner to outer ring colors indicate cluster, origin region, sample isolation type, and data sources, respectively. These rings are further subdivided. Then the phylogenetic tree presented by the actual branch lengths and labeled the bootstrap values is shown in detail in Fig. S2.

The phylogenetic information and subclades architecture derived from genome data of globally representative *E. meningoseptica* strains were used to establish a comprehensive genotyping system. Figure 2 shows a maximum likelihood phylogenetic tree based on SNPs. Phylogenetic analyses primarily indicated that the Chinese *E. meningoseptica* isolates we collected were distributed throughout this framework. By sampling across the continent with a structure tree, we observed a substantial level of genetic diversity from different sources and countries (Fig. 2). This tree is divided into four main branches, each containing species from different regions and sources. The majority of clinical and environmental isolates resided on distinct branches of the SNP tree, with clinical isolates showing greater diversity than environmental bacteria. Among these *E. meningoseptica* strains, there is no compact phylogenetic group with special characteristics, indicating that the evolutionary history of the isolates has no extraordinary connection with the sample origin, source, and region. Although extensive genomic diversity exists in the circulating *E. meningoseptica* population, we found that all cluster 3 strains were only found in China, except for one isolate from an unavailable region. Interestingly, the map shows that clinical cerebrospinal fluid cultures of *E. meningoseptica* were only found in cluster 4 (Fig. 2).

**Fig 3:**
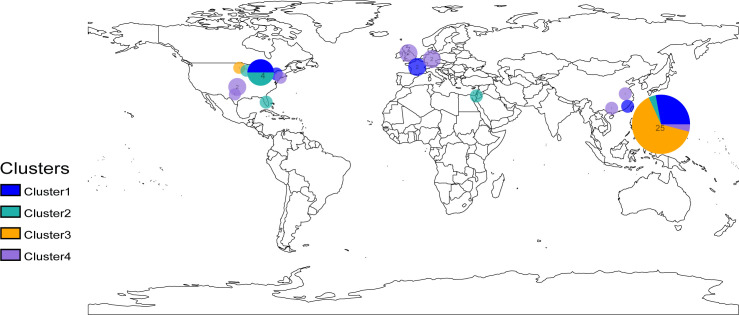
The geographical distribution of global origin *E. meningoseptica* populations. Map displaying the locations of the field sites where *E. meningoseptica* isolates were obtained for this study. Each colored circle indicates the isolates from a specific city/country, with a radius in proportion to the sample size. The pie charts indicate the proportion of the main genotypes isolated. The colors in the circles and the note below correspond to the population branches as shown in Fig. 2. The map was generated using R software (version 3.5.3) in conjunction with the rworldmap package.

Our study investigated the global distribution of *E. meningoseptica* genomes based on genotypes and strain regions (Fig. 3). This analysis combined isolates from the same clade and country into a single representative circle (Fig. 3). The results revealed that *E. meningoseptica* bacteria have been transmitted to numerous countries worldwide, particularly China and the USA. Different economic levels in countries are associated with multiple genotypes, indicating a high level of genetic diversity. However, almost all cluster 3 isolates (blue) are found exclusively in China (Fig. 2 and 3). Intriguing, *E. meningoseptica* is present in Asia, Europe, and North America, but not in South America, Africa, or Oceania (Fig. 3). Additionally, the distribution of *E. meningoseptica* strains is closely related to latitudes (Fig. 3). This pathogenic bacterium has the potential to spread horizontally between countries, posing a global threat.

### Emergence timeline of *E. meningoseptica* and genetic clusters

**Fig 4:**
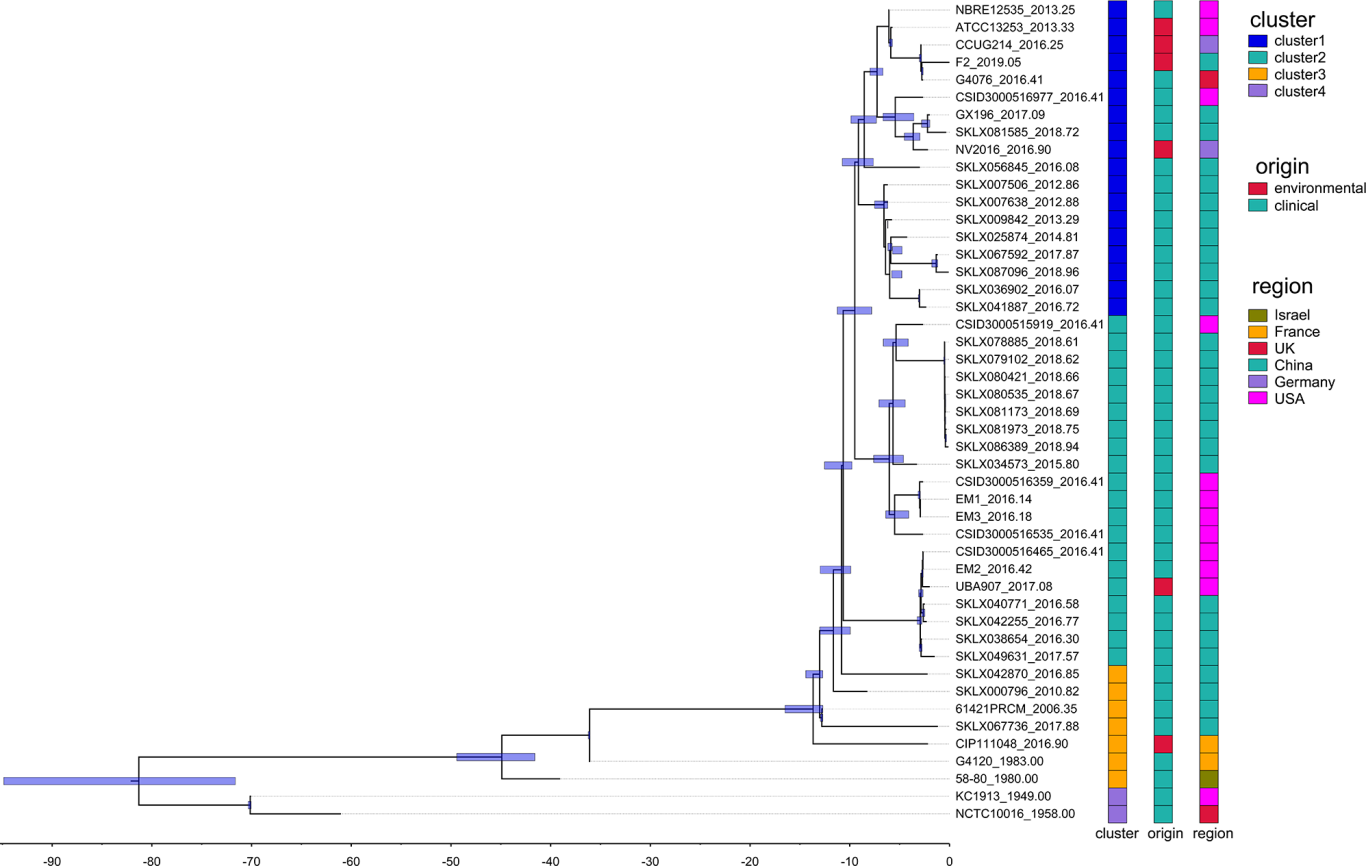
Temporal analysis on *E. meningoseptica* isolates. Time-calibrated Bayesian phylogeny is constructed using BEAST v.2.5.2. Horizontal bars in light purple, centered on nodes, represent 95% highest probability density (HPD) values. Time is indicated on the *x*-axis; vertical dashed lines correspond to the first day of the indicated year. Isolate labels (shown as vertical bars on the right) are color-coded to indicate cluster, origin, and source region.

No phylogeographic reconstruction has been performed for *E. meningoseptica* bacteria. Therefore, we conducted temporally resolved phylogenies using Bayesian evolutionary analysis by sampling trees. Initially, we tested the correlation between root-to-tip distances and isolation dates of *E. meningoseptica* isolates. The tip-randomization test in the Bayesian analysis demonstrated a significant temporal signature (Fig. S4), indicating that the strain continued to diversify in a measurable way over the course of evolution. The BEAST analysis estimated that the most recent common ancestor of the *E. meningoseptica* clades dated back 80 years ago [95% highest probability density (HPD) interval, 1929–1949]. In the recombination-free *E. meningoseptica* BEAST tree (Fig. 4), the isolates were divided into three well-supported lineages, which diverged around 1938, 1974, and 2005 respectively. In the most recent lineage, all isolates formed several distinct and strongly supported subclades, each belonging to its own subclade (Fig. 4). Rather than being driven by a single clade within the Chinese origin lineage, the entire lineage has increased in prevalence since its emergence in the 2000s. Our collection of *E. meningoseptica* also demonstrates this through an analysis of isolation dates against its phylogeny (Table S1). Based on the analysis results, the USA may be the most likely source of these pathogenic *E. meningoseptica* strains, which emerged *de novo*, and the source of four major pathogenic subtypes. It has spread multiple times to other nearby countries. However, further epidemiological evidence for intercontinental transmission needs to be observed.

**Fig 5:**
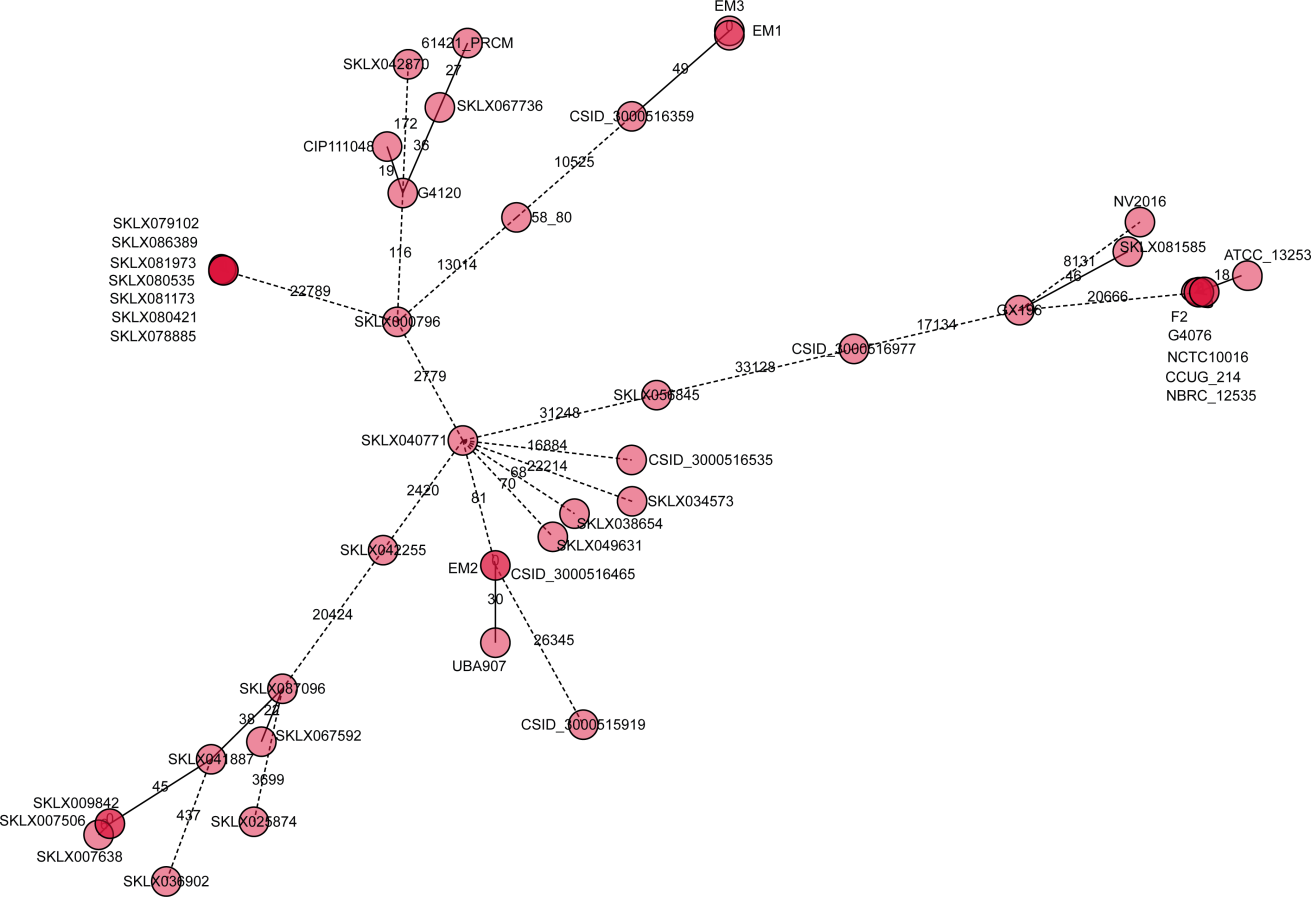
Genetic distances within *E. meningoseptica* isolates clusters. Genetic distances were estimated using the minimum spanning tree method. Isolates are depicted as nodes, labeled with sample names. Lines connecting *E. meningoseptica* isolates represent genetic SNP distances, with the length of the lines indicating the magnitude of the distance. Nodes in light red color containing multiple strain numbers indicate the presence of multiple isolates with genome sequences that are ≤10 SNPs apart. Dashed lines represent larger SNP distances (not to scale) with numbers indicating the SNP difference.

In order to determine if *E. meningoseptica* genotyping is related to genuine transmission, we assessed the proportion of isolates genetically linked to 10 or fewer SNPs. After typing 47 isolates, we identified 33 loci (Fig. 5). The expected star-like pattern, indicating a potential-spreader, was observed in all pathogenic bacteria. In a putative transmission network among individuals sharing clustered strains, three individuals (SKLX800796, SKLX087096, and GX196) appeared to have possible epidemiological links (Fig. 5), suggesting the presence of a super-spreader. Nevertheless, more clinical and epidemiological information is needed to support these findings. The results showed that seven isolates (SKLX079102, SKLX086389, SKLX081973, SKLX080535, SKLX081173, SKLX080421, and SKLX078885) formed a circle (Fig. 5). Despite being isolated from different patients, this finding is consistent with the idea that sharing genomic clustered strains originated from the same hospital. Surprisingly, five transnational strains (F2, G4076, NCTC10016, CCUG_214, and NBRC_12535) also formed a circle (Fig. 5). Although there is no epidemiological evidence, these isolates share almost the same SNP, indicating they may have originated from the same pathogenic bacteria ancestor. All in all, the putative transmission networks suggest that *E. meningoseptica* bacteria could spread within the same hospital, province, or even across borders, potentially causing a severe nosocomial outbreak of infections.

### Antimicrobial susceptibility and resistance determinants

The antimicrobial susceptibility results are presented in Table S2. The isolated strains of *E. meningoseptica* exhibited varying levels of multidrug resistance to the tested antibiotics. Corresponding to reports, they are resistant to several commonly used antibiotics, including cephalosporins, penicillins, aminoglycosides, fluoroquinolones, tetracyclines, polypeptides, and even carbapenems (Table S2). In all isolates, piperacillin, piperacillin-tazobactam, ceftazidime, cefepime, imipenem, meropenem, aztreonam, gentamicin, amikacin, tetracycline, and trimethoprim-sulfamethoxazole were resistant. By contrast, minocycline and doxycycline showed no resistance. Furthermore, levofloxacin and rifampin also demonstrated high rates of *in vitro* activity against clinical isolates of *E. meningoseptica* (Table S2). Minocycline or doxycycline may be promising candidates as the drugs of choice for treating *Elizabethkingia* infections. Therefore, further clinical studies may be needed to determine their potential role in treating *E. meningoseptica* infection.

**Fig 6:**
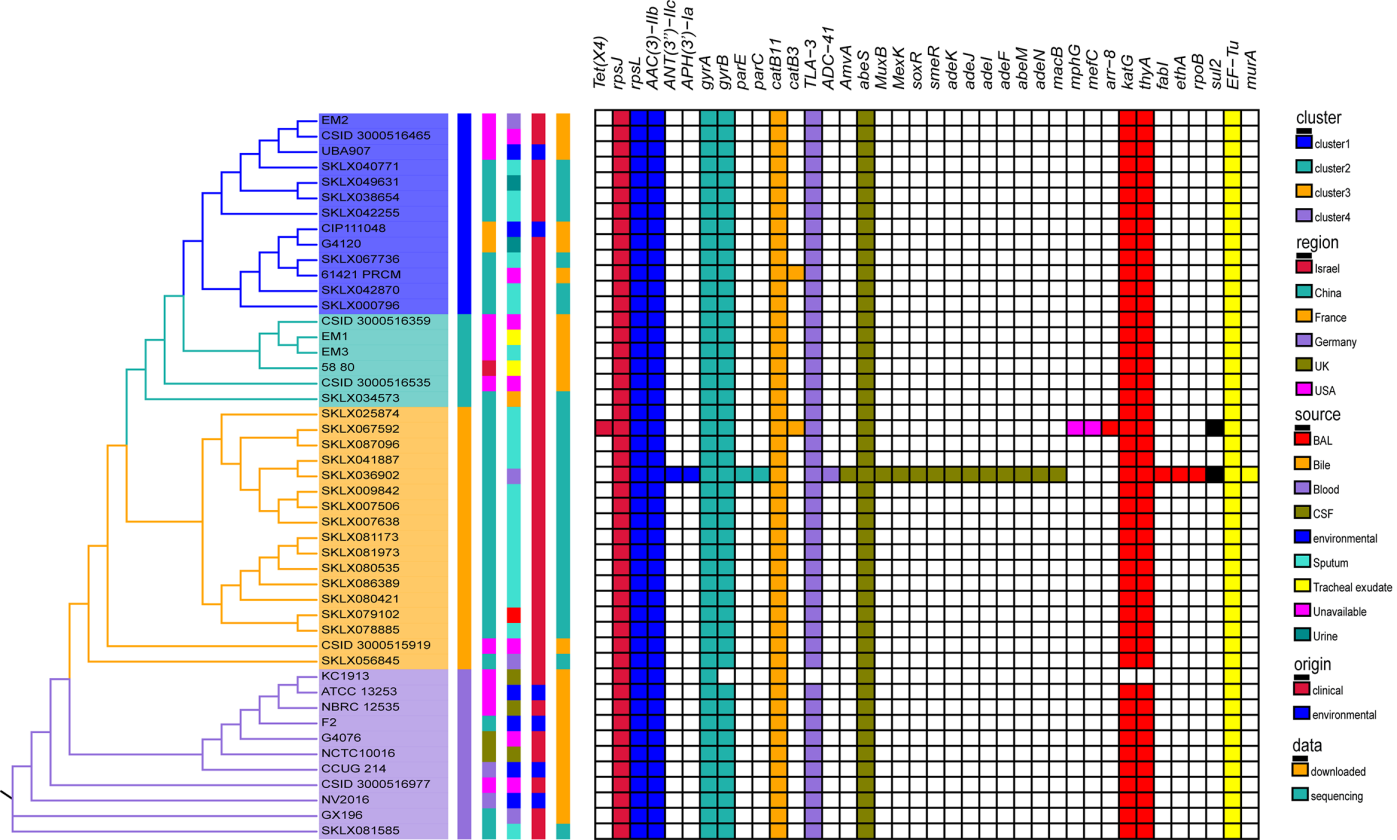
Phylogenetic distribution of genes encoding antibiotic resistance. Maximum likelihood phylogenetic tree of 47 *E. meningoseptica* isolates from this study with corresponding metadata including genotype, location, and antimicrobial resistance genes. The right side of the phylogenetic tree displays five bars representing cluster, region, sample type, origin, and data sources, respectively. The colors in the left-hand columns correspond to those in Fig. 2. Antibiotic family groups resistance genes in a clade (right-hand side). Different colors represent different types of resistance genes: from left to right are tetracycline, aminoglycoside, fluoroquinolone, chloramphenicol, beta-lactams, efflux pump, macrolide, antituberculosis drug resistance, sulfonamide, and other types.

Across all isolates of *E. meningoseptica* genomes, antibiotic resistance genes were mapped (Fig. 6). More than 10 types of resistance genes (out of 38 specific genes) were detected, including tetracycline resistance gene, aminoglycoside resistance gene, fluoroquinolone resistance gene, beta-lactam resistance gene, and resistance efflux pump (Fig. 6). However, no special mutant genes were found after screening. Seven resistance genes (*rpsJ*, *rpsL*, *aac (3)-IIb*, *gyrA*, *catB11*, *abeS*, and *EF-Tu*) were present in all strains' genomes (Fig. S5), suggesting that the *E. meningoseptica* species has low intrinsic sensitivity to these antibiotics. Notably, a blood isolate SKLX036902 exhibited half as many resistance genes as other strains, which requires further experimental verification (Fig. 6; Fig. S5). Overall, there were few variations in antimicrobial resistance genotypes among different isolates. Furthermore, the results indicated no significant lineage specificity, even when specific lines showed minimal variation (Fig. 6). The distribution of resistance genes on the map did not correlate with the geographic origin or source of the strain. The presence of antibiotic-resistance genes revealed a substantial homogeneity among *E. meningoseptica* isolates. With the exception of four isolates, all *E. meningoseptica* strains shared the same antibiotic resistance determinants (Fig. 6), indicating that the multi-drug resistance characteristic may be inherent and inherited from common ancestors.

### Virulence genes and potential pathogenicity

**Fig 7:**
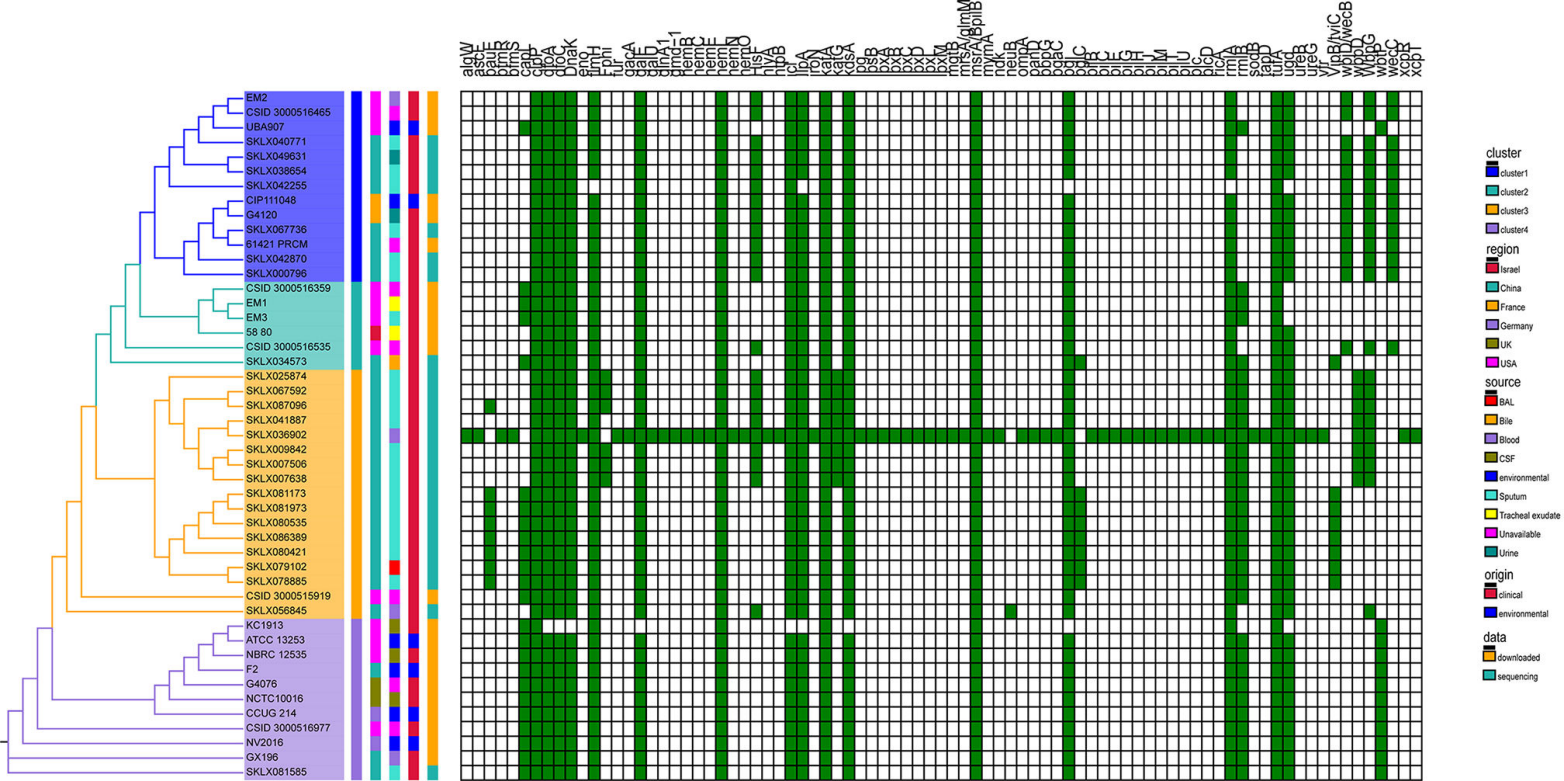
The virulence genes distribution within global *E. meningoseptica* isolates. The left-hand side is the maximum likelihood SNP phylogenetic tree, with corresponding bar and color annotations as shown in Fig. 6. The right-hand side displays the distribution of virulence genes. All recognized virulence genes are shown as green (present) or white (absent).

The genomes of all 47 global *E. meningoseptica* isolates were screened for known genetic determinants of virulence factors. Over 80 different acquired virulence genes were detected (Fig. 7). These virulence genes mainly include capsule polysaccharide synthesis, core polysaccharides and lipid A lipopolysaccharide (LPS) synthesis, Type IV pili biosynthesis, biofilm synthesis, and heme biosynthesis (Fig. 7). Six gene-related heme biosynthesis and utilization (*hemB*, *hemC*, *hemE*, *hemL*, *hemN*, and *hemO*) were identified, partially explaining the blood infection pathogenic ability of *E. meningoseptica* bacteria. For example, clinical bacteremia infections caused by this bacterium have been reported. Although *clpP*, *hmeL*, *katA*, *msrA*/*bpilB*, and *tuFA* genes were recognized in all studied strains (Fig. S6), this shows a partial degree of pedigree specificity with minimal variation within a specific main strain. Overall, strain virulence genes have little correlation with the source of strain samples and geographic location (Fig. 7). Furthermore, there is little difference in virulence genes between clinical and environmental strains, potentially suggesting the inherent pathogenicity of such bacteria. Notably, a Chinese *E. meningoseptica* isolate exhibited significantly more virulence genes than other strains (Fig. 7). Consistent with drug resistance gene analysis, the blood-isolated strain SKLX036902 displayed not only more drug resistance genes but also a higher number of virulence genes, making it particularly valuable and deserving of further study.

## DISCUSSION

*E. meningoseptica* is ubiquitously distributed in nature, including soil, water, and hospitals. Recently, it has been increasingly recognized as a pathogen that can cause nosocomial infections in immunocompromised individuals. In this study, we collected over 20 clinical isolates of *E. meningoseptica* around a 10-year period and conducted complete genome sequencing and comparative genomics analysis. Whole genome sequencing and analysis have become important tools in studying pathogens, thanks to advancements in molecular biology and biotechnology. Previous research has mainly focused on case studies and the susceptibility of *E. meningoseptica* to antibacterial agents. However, there is limited available genomic information on this bacterium. To the best of our knowledge, this is the first comprehensive analysis of the evolutionary relationships and comparisons among *E. meningoseptica* bacteria using extensive genome sequencing.

According to a previous study, diagnosing uncommon non-fermenting bacteria poses difficulties (40, 41). The current methods for distinguishing between *Chryseobacterium indologenes* and *E. meningoseptica* lack reliability and consensus (40, 41). Traditional methods are challenging for identifying *C. indologenes* and *E. meningoseptica*. Previous studies have shown that these techniques are not highly effective in distinguishing between *Chryseobacterium* and *Elizabethkingia* species (40 – 43). Among automated phenotypic methods, VITEK 2, MALDI-TOF MS, MALDI-BD (MALDI-TOF BioTyper), Phoenix 100 ID/AST, and API (Analytical Profile Index) are used to identify *E. meningoseptica* isolates. However, due to limited database coverage, other *Elizabethkingia* species are often misidentified as *E. meningoseptica* (40, 42, 43). Especially, the API/ID 32 v3.1 system has an identification rate of less than 30% for *Elizabethkingia* species (42). Recent papers have also shown that *Elizabethkingia anophelis* is frequently misidentified as *E. meningoseptica* using current commercial identification systems (43 – 45). Therefore, it is recommended to modify the method for discriminating *E. meningoseptica* from *Chryseobacterium gleum* and other *Elizabethkingia* species and to expand the database coverage for *E. anophelis* in the discussed microbial identification systems. Generally speaking, 16S rRNA gene sequencing is the preferred method for strain identification. While this traditional approach can successfully identify most novel strains, it may encounter inaccuracies when dealing with rare strains. Our investigation revealed the presence of 35 additional *E. meningoseptica* genomes, including raw data in Sequence Read Archive (SRA) in the NCBI database, apart from the strains we obtained. After conducting an ANI analysis, we found that the values of 13 isolates were significantly low (less than 80%) to be considered as *E. meningoseptica* or even belonging to the same genus (Fig. S7). Interestingly, the ANI value of isolate 5453STDY7605978 among these 13 strains was relatively low, at 62.73%, indicating a potential error in either the sequencing or submission process of the genome (Fig. S7). To address these issues, it is recommended to design new specific primers for species identification through 16S rRNA gene sequencing. These findings emphasize the importance of obtaining ANI values prior to conducting a genome analysis of rare and novel pathogenic bacteria.

Several previous studies have shown that *Elizabethkingia* species isolated from different geographic regions are susceptible to different antibiotics and exhibit complex antimicrobial resistance characteristics (15, 45). Our study results indicate that the geographic distribution of *E. meningoseptica* resistance genes is not linked. It is intriguing to consider how the biofilm may interpret this phenomenon and the multidrug resistance mechanism of *E. meningoseptica* species. A recent study demonstrated that *E. meningoseptica* bacteria have the ability to form biofilms and adhere (46). Our investigation revealed a great number of genes associated with biofilm formation. Biofilm has become a prominent topic of discussion in the context of antibiotic resistance in pathogenic bacteria in recent years. Chloramphenicol is not typically used to treat internal bacterial infections nor has it been investigated for *Elizabethkingia* species. Remarkably, chloramphenicol has been found to be a potent antibiotic capable of destroying various bacteria that can cross the blood-brain barrier, and it also possesses anti-biofilm properties (47). Chloramphenicol could potentially be utilized to treat meningitis infections caused by *E. meningoseptica*.

We hypothesized that many *E. meningoseptica* isolates were connected through transmission networks, as they were less than 10 SNPs apart from one another, regardless of the origin, whether it was a person or a different nation. This situation is particularly interesting. This discovery is of great importance as it implies that *E. meningoseptica* could be transmitted within a hospital, across borders, and even cause notorious outbreaks of infection. It has been challenging to find epidemiological evidence supporting the existence of direct genetic super-spreaders. Since the mode of transmission of *E. meningoseptica* is unclear and the organism is resistant to multiple classes of antimicrobials, treating an infection caused by this organism is a daunting task. This typical pattern of antimicrobial susceptibility can hinder the selection of the most suitable medications. In contrast, there is a scarcity of antimicrobial data for *E. meningoseptica*, and the results of susceptibility testing can vary depending on the method used (14). Given the difficulty in treating this organism with antimicrobials, it is advisable to use susceptibility testing to guide the choice of treatment. It has been suggested that patients who require long-term acute care with mechanical ventilation may be a significant source of transmission for the multi-drug resistant pathogen, *E. meningoseptica*, following the outbreak description (5).

There are several limitations to our present study. Firstly, it is a retrospective and single-center investigation, as our *E. meningoseptica* strain was predominantly obtained from a tertiary medical center. Secondly, the sample size of this study was limited, despite the inclusion of samples from 2010 to 2019, and the sources were diverse, which may not effectively represent the broader distribution in China. To address these limitations, it is necessary to sequence a larger number of *E. meningoseptica* strains from China and other regions worldwide. Furthermore, it is important to note that no minimum inhibitory concentration (MIC) cut-off values have been established by the Clinical and Laboratory Standards Institute (CLSI) and European Committee on Antimicrobial Susceptibility Testing for *E. meningoseptica strains*. Therefore, this article relies on published literature to develop interpretive criteria for MIC (48, 49).

In summary, *E. meningoseptica* has recently been identified as a pathogen that can cause severe and potentially lethal infections in humans. Variations in resistance and virulence genes further support the existing evidence of the natural resistance and pathogenic nature of *E. meningoseptica*. Our research emphasizes the possibility of a significant outbreak of *E. meningoseptica* in hospitals and its potential to spread internationally. It is recommended that increased genomic monitoring be carried out to gain a deeper understanding of the dynamics of *E. meningoseptica* populations.

## MATERIALS AND METHODS

### Sampling and susceptibility interpretation

To investigate *E. meningoseptica* and obtain samples for genome sequencing, we conducted a survey to screen *E. meningoseptica* isolates from the Strain Sample Bank of the First Affiliated Hospital of Zhejiang University School of Medicine. These isolates were collected between January 2010 and April 2019 for routine clinical purposes. Initially, we identified these isolates using MALDI-TOF MS (Bruker Daltonics, USA) and stored them as glycerol stocks at −80°C until further use. Ultimately, we identified 25 *E. meningoseptica* isolates, which were obtained from various sources including sputum (20 isolates), blood (2 isolates), urine (1 isolate), bile (1 isolate), and bronchoalveolar fluid (1 isolate). In addition to the strains we collected, all available NCBI *E. meningoseptica* genomes at the time (June 2020) of our study were included in this work. Further information can be found in Table S1.

The agar dilution method was used to determine the MIC in accordance with the guidelines set by the CLSI (50). The CLSI criteria for “other non-Enterobacteriaceae” were used to interpret the results of the agar dilution method, except for tigecycline, vancomycin, and rifampin. For tigecycline susceptibility testing, the US Food and Drug Administration breakpoints for Enterobacteriaceae were applied (MIC: resistant, ≥8 mg/L; intermediate, 4 mg/L; susceptible, ≤2 mg/L) (48, 49). The susceptibility breakpoints for Enterococcus species were determined based on the CLSI standards for vancomycin and rifampin (50).

### Genome sequencing

Briefly, the strains were spread on Mueller-Hinton agar plates (Becton Dickinson, Sparks, MD, USA) and incubated overnight at 37°C. After inoculation of a new colony, it was cultured overnight at 37°C on a shaker with Mueller-Hinton broth (Oxoid, UK). Genomic DNA was prepared using the Gentra Puregene Yeast/Bact. Kit (Qiagen, Germany) following the manufacturer’s instructions. The Illumina NEBNext Ultra DNA Library Prep Kit (NEB, USA) was utilized for the preparation of sequencing libraries. Library size distribution and quantitation were analyzed using real-time PCR and the Agilent 2100 Bioanalyzer. Whole genome sequencing was conducted using Illumina NovaSeq to generate 150 bp paired-end reads. Initial assembly was performed using three different genome assembly software (SOAP denovo, SPAdes, and Abyss), followed by integration of the results using CISA software.

### Genomic data analysis

To reconfirm the presence of these emerging species, we utilized the FastANI tool to calculate the ANI values of all available *E. meningoseptica* genomes (51). For gene repertoire analysis, CMG-Biotools package was used to generate column distributions for the pan-genome and core-genome (52). The RedDog phylogenomics pipeline was utilized for read mapping, SNP calling, and preliminary filtering (https://github.com/katholt/RedDog). The identified SNPs among all *E. meningoseptica* strains were combined, and a maximum likelihood phylogenetic tree was constructed using PhyML (53). The root of the evolutionary tree was determined based on the random median value. For detailed information on the construction methods of the SNPs phylogenetic tree, please refer to our previous study (54). The geographical distribution of the global graph was analyzed using the rworldmap package in R software (version 3.5.3).

Temporal phylogenetic analysis and dating of *E. meningoseptica* isolates were conducted using BEAST v.2.5.2, utilizing the core SNPs alignment (55). To assess the temporal signal, molecular tip-randomization analyses were performed using the R package TipDatingBeast, based on 10 samples with reshuffled dates (56). The model selection process involved a nested sampling method and three clock models (relaxed exponential, relaxed log-normal, and strict clock models), each combined with constant, exponential, and coalescent Bayesian skyline population models (57). A detailed model description of timeline reconstruction and the most recent common ancestor determination could be found in our previous research (54).

Genetic distances were estimated using the minimum spanning tree method. *E. meningoseptica* strains that were ≤10 SNPs apart were categorized as the same cluster. The CARD database (58) and the VFDB protein Set B database (59) were used to screen for acquired antimicrobial resistance genes and virulence genes distribution among all global *E. meningoseptica* isolates, respectively. Both filtering parameters were set as follows: protein identity > 50%, query coverage > 50%, subject coverage > 50%, match length > 100 amino acids, and identical > 100 amino acids. The distribution of resistance genes and virulence genes was visualized using R software (version 3.5.3) through the creation of a heatmap.

## Data Availability

Every genome sequence assembled for the 25 newly sequenced *E. meningoseptica* strains has already been deposited in the GenBank of NCBI under project no. PRJNA736797. Genomes are available by corresponding sequence accession numbers. A summary of all *E. meningoseptica* genome data used in this study can be found in Table S1.

## SUPPLEMENTAL MATERIAL

Supplemental figures spectrum.01602-23-s0001.pdf Fig. S1 to S7.

Table S1 spectrum.01602-23-s0002.xlsx
*E. meningoseptica* isolates and comparative strains included in the study.

Table S2 spectrum.01602-23-s0003.xlsx Antimicrobial MICs of 25 *E. meningoseptica* isolates determined using agar dilution.

ASM does not own the copyrights to Supplemental Material that may be linked to, or accessed through, an article. The authors have granted ASM a non-exclusive, world-wide license to publish the Supplemental Material files. Please contact the corresponding author directly for reuse.
