# Supplementary material for: Probability of outbreaks and cross-border dissemination of the emerging pathogen: a genomic survey of Elizabethkingia meningoseptica
Source: Microbiol Spectr. 2023 Oct 10;11(6):e01602-23. doi: 10.1128/spectrum.01602-23 (PMC10714787; doi:10.1128/spectrum.01602-23)
Supplement: Supplemental figures — Fig. S1 to S7. [file spectrum.01602-23-s0001.pdf]

# Supplemental Figures

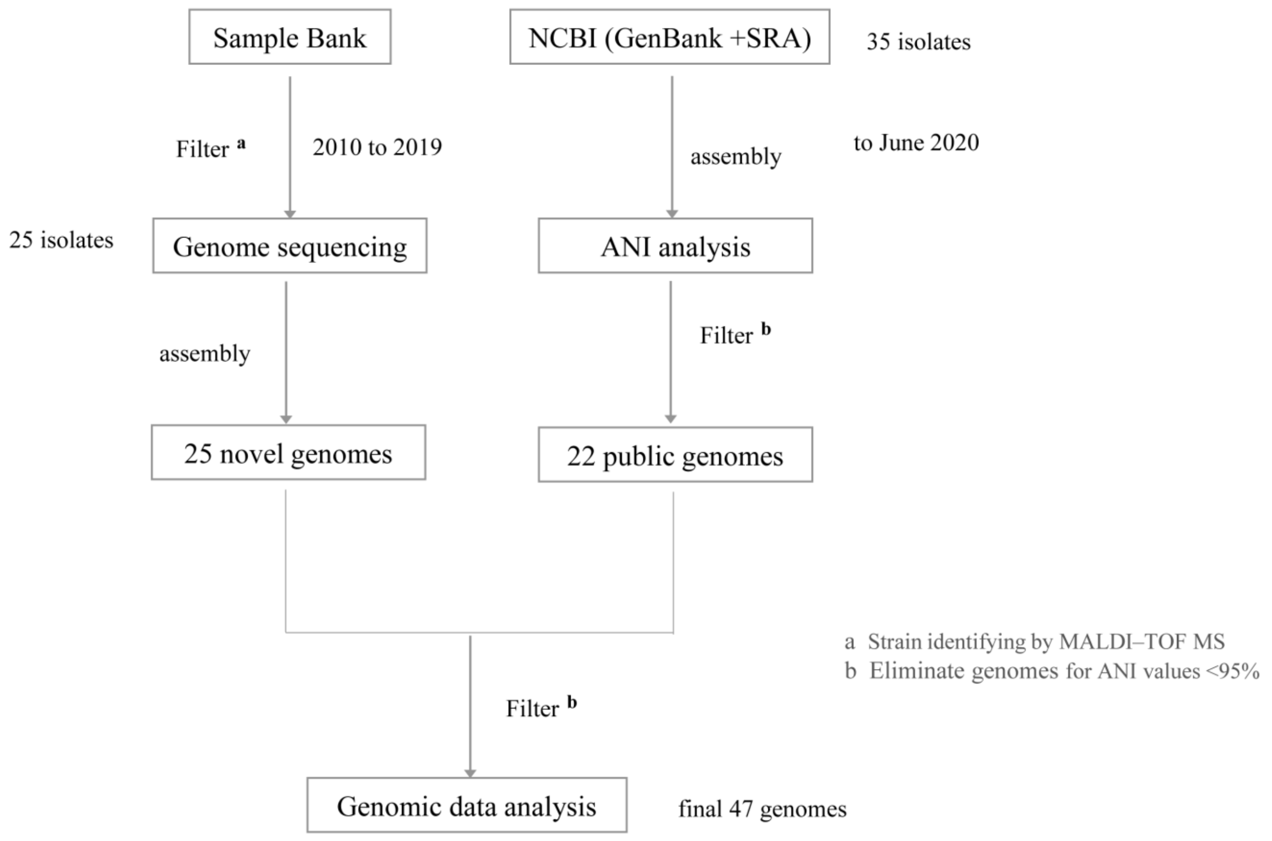


**Figure S1. The workflow of the establishment of genomic analysis among global *E. meningoseptica* species.** ANI: the average nucleotide identity; MALDI-TOF MS: matrix-assisted laser desorption ionization time-of-flight mass spectrometry systems.

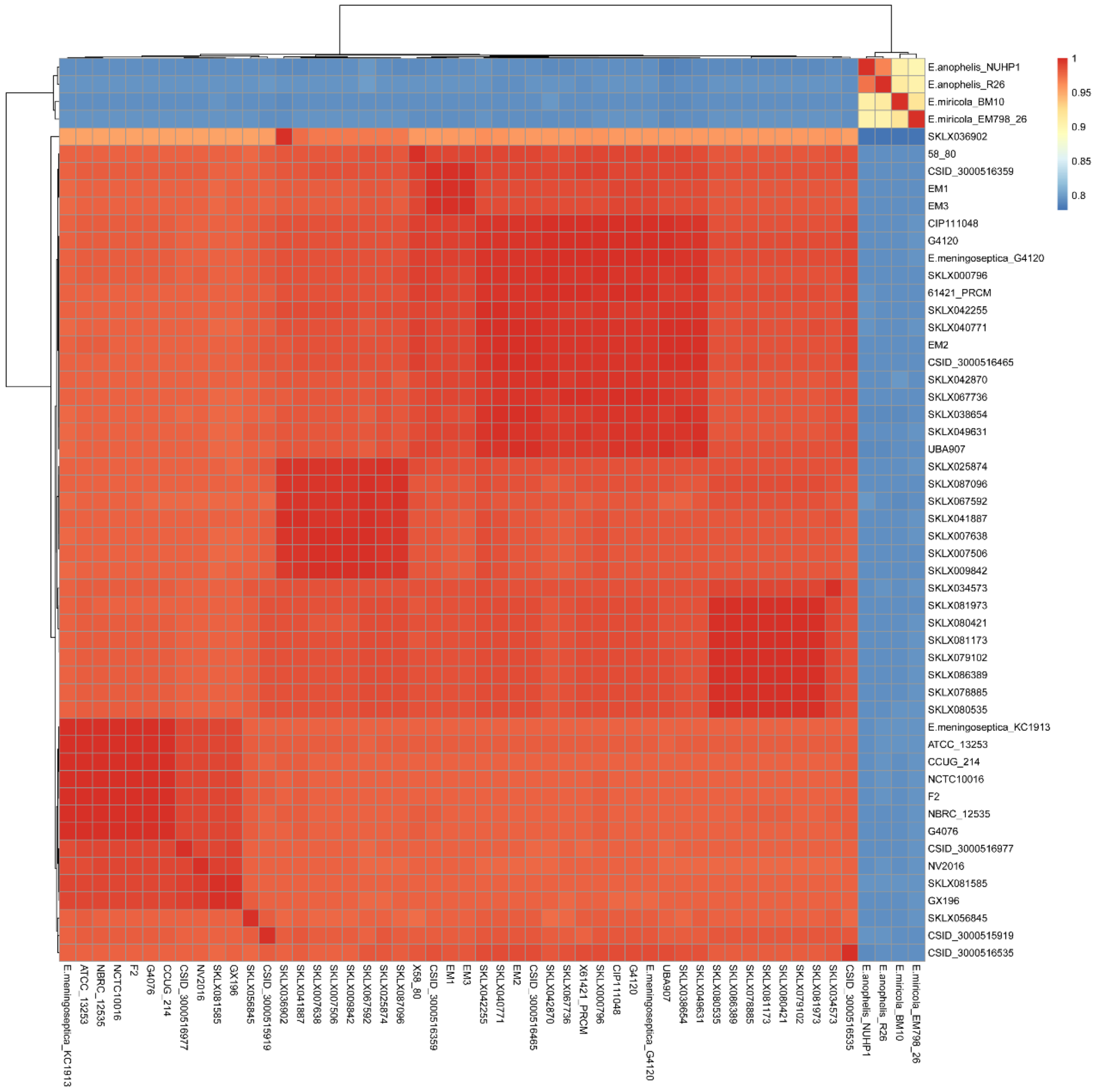


**Figure S2. The ANI value heatmap among representative *E. meningoseptica* species.** A total number of 51 strain are presented on the heatmap, including our collected 25 *E. meningoseptica*, downloaded 22 *E. meningoseptica*, and other four referenced species (*E. miricola* and *E. anophelis*). The ANI values among all *E. meningoseptica* species are >95%.

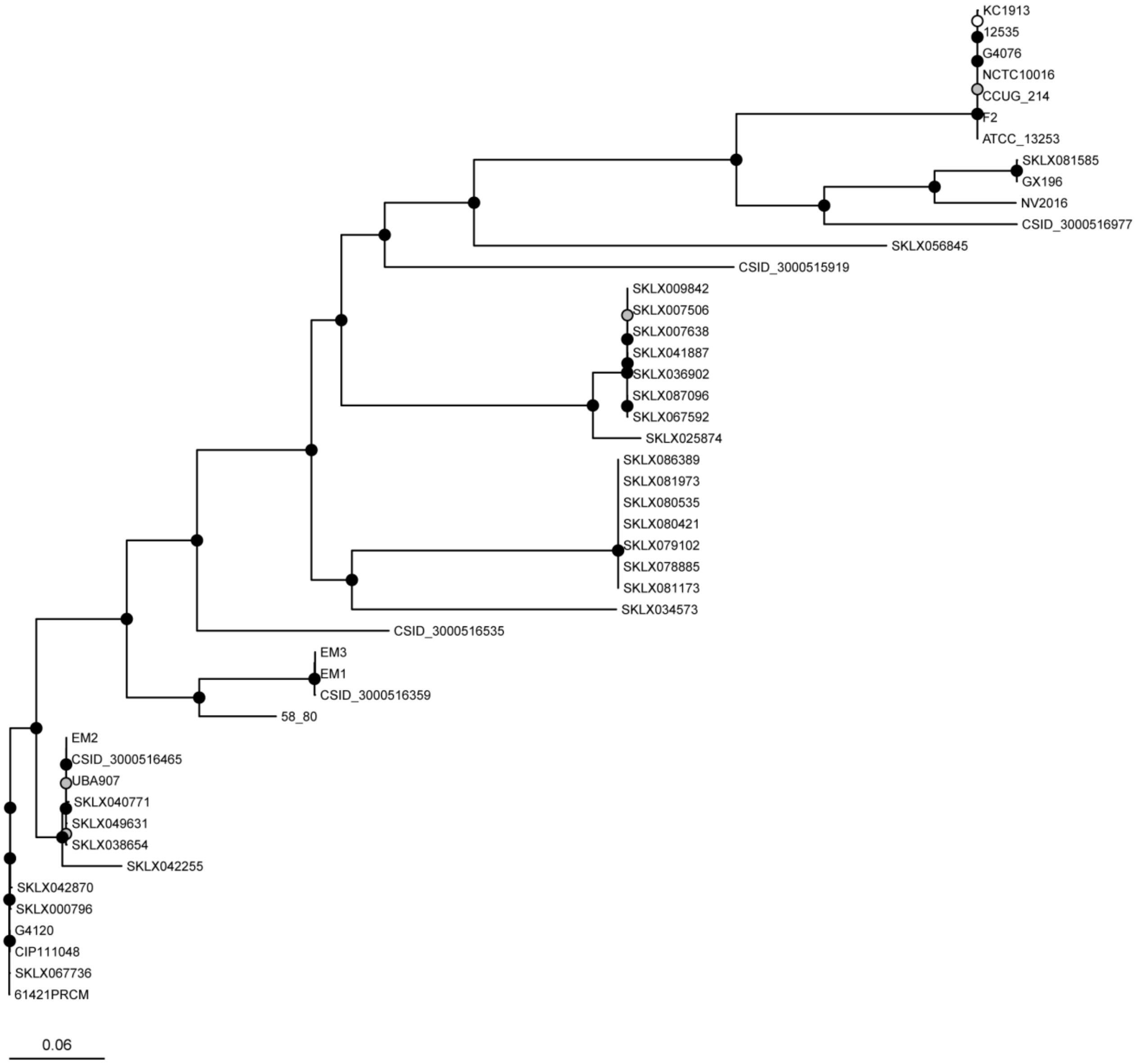


**Figure S3. The phylogenetic SNP tree displaying the actual structures and branch lengths.** The colored dots indicate the corresponding bootstrap percentage values: black dots represent bootstrap values ≥ 90; gray dots represent bootstrap values ≥ 70 and < 90; white dots represent bootstrap values < 70.

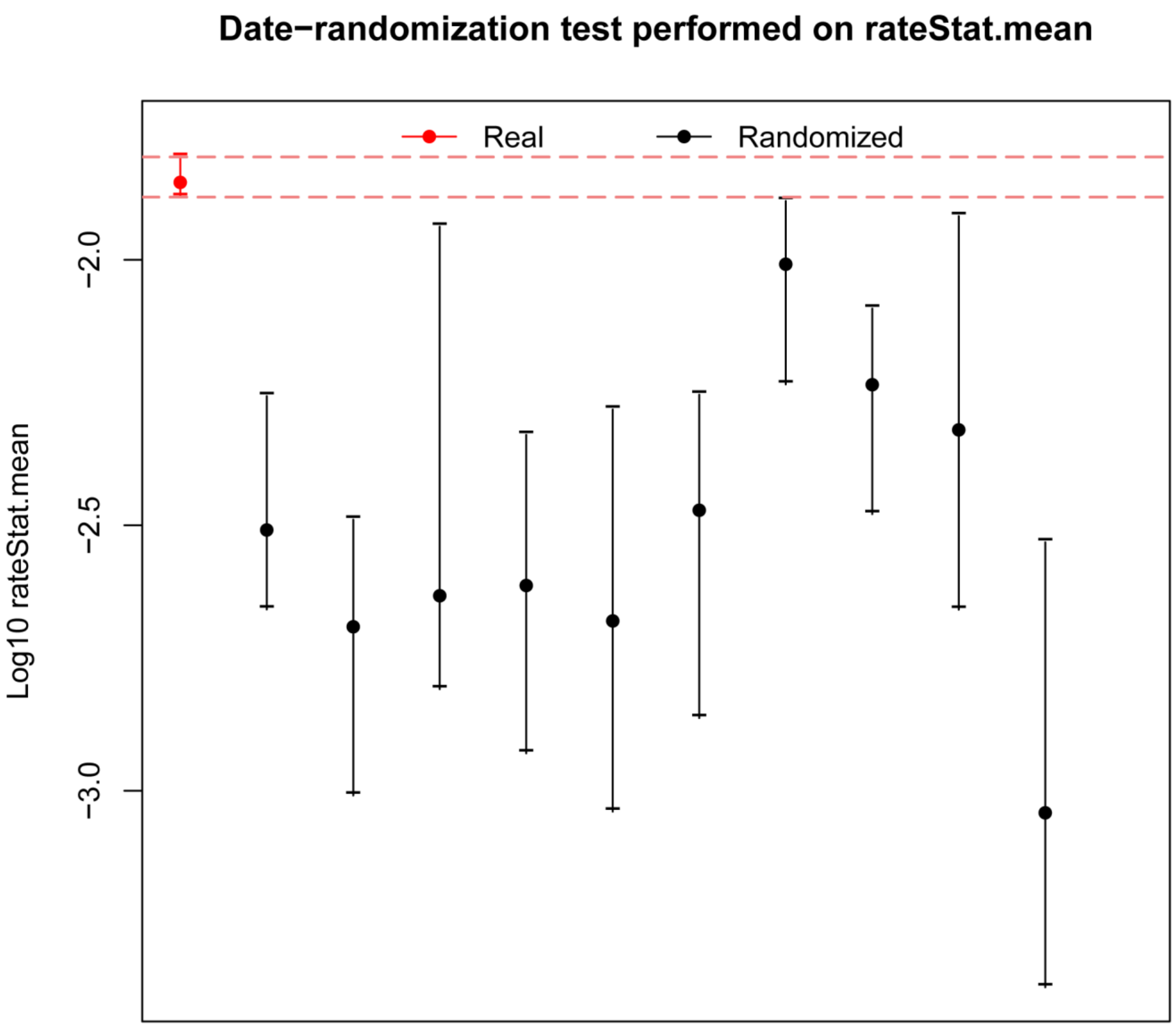


**Figure S4. BEAST tip-randomization test for temporal signal.** The logarithm (base 10) of the posterior mean (circles) and the 95% highest posterior density (bars) of the estimated randomized replacement rate using *E. meningoseptica* data (top, red) and ten tip dates (bottom, light black).

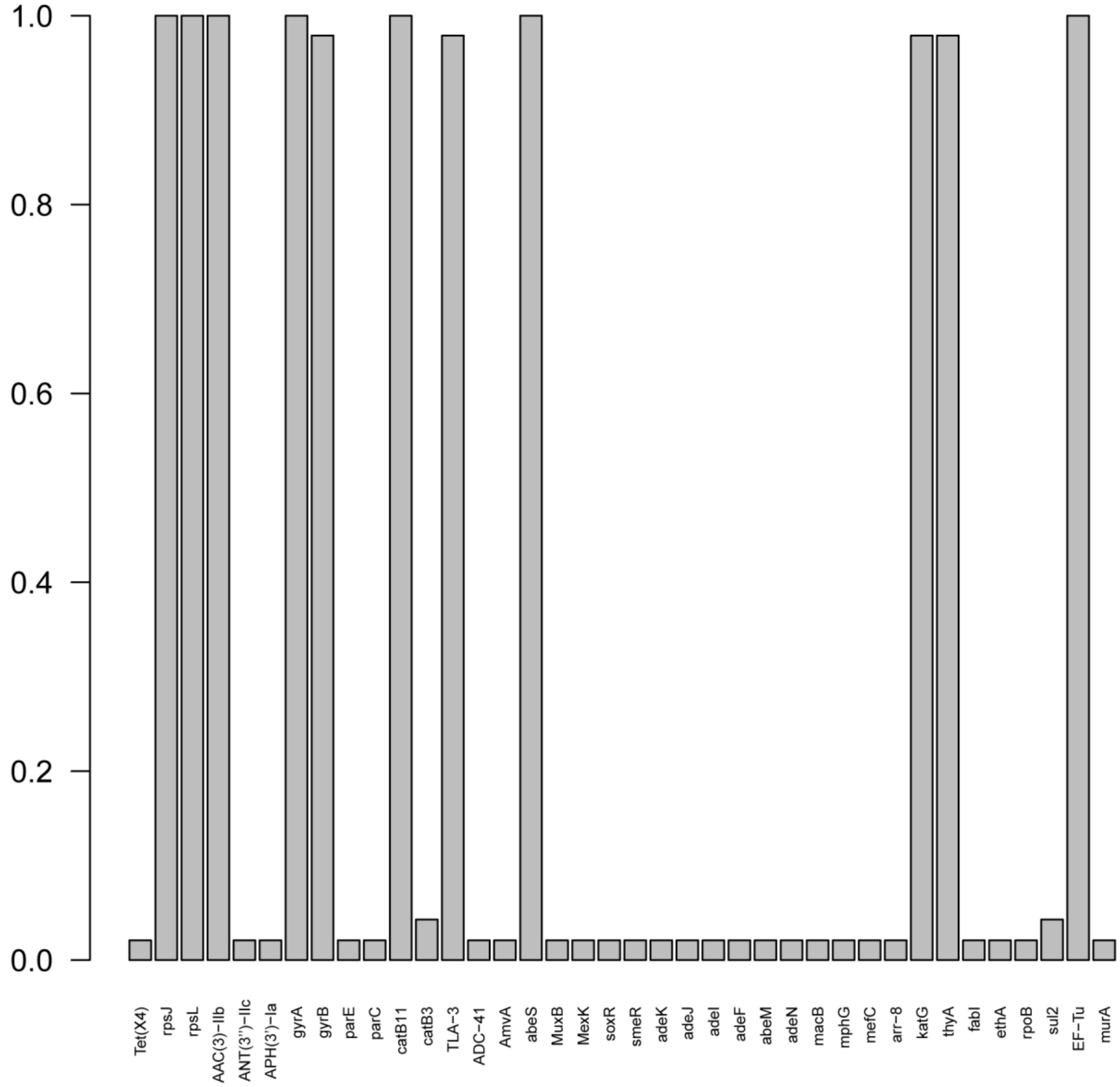


**Figure S5. Identification frequency of each antimicrobial resistance gene.** The x-axis means the distribution of recongnized resistance genes, while the y-axis represents the proportion of individual number containing the gene. The distribution of antimicrobial resistance genes aligns with Figure 6.

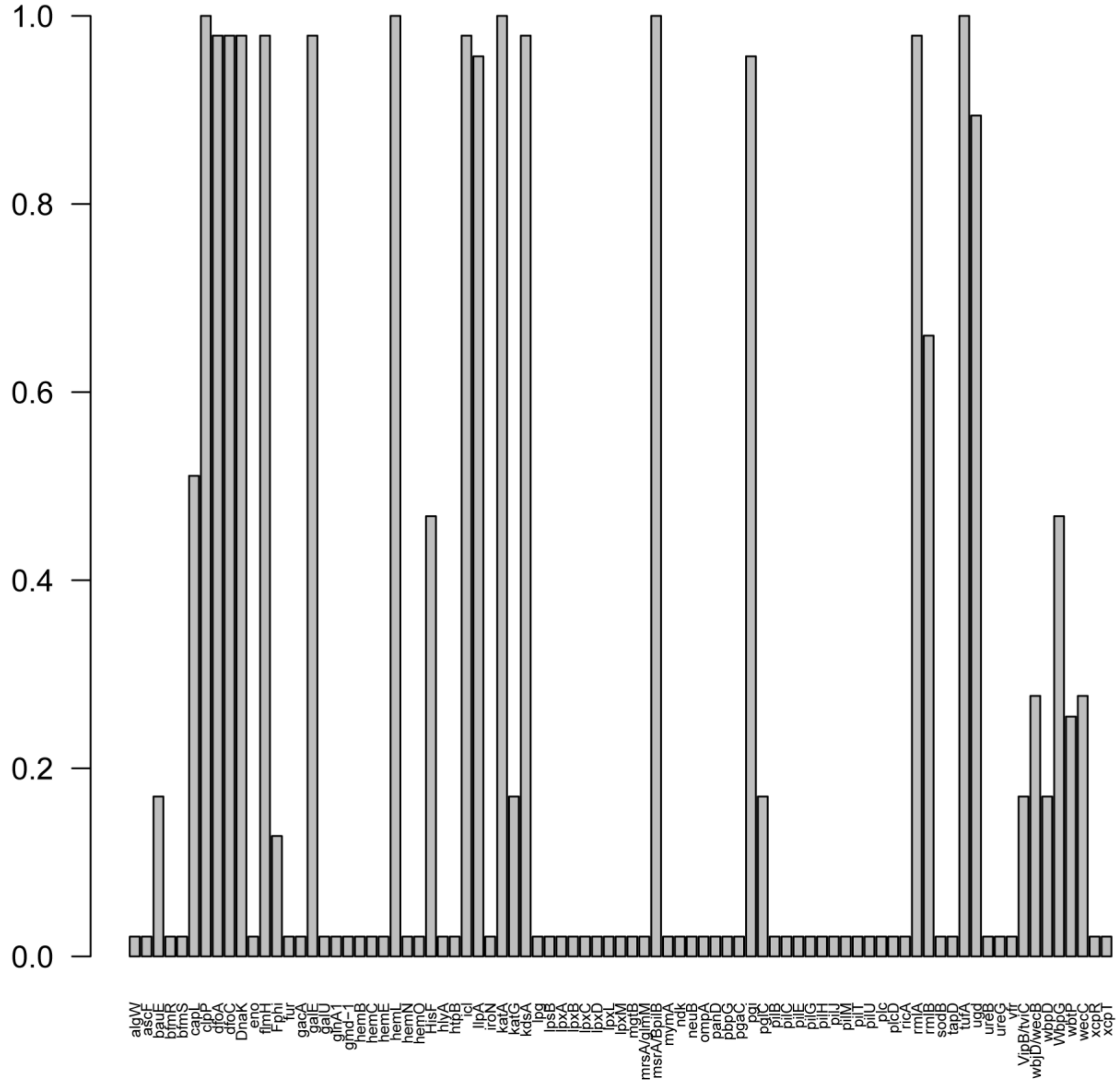


**Figure S6. Detection frequency of each corresponding virulence gene.** The x-axis represents the distribution of virulence genes, while the y-axis indicates the related proportion of strain numbers who containing the gene. The distribution of virulence genes is corresponding to the Figure 7.

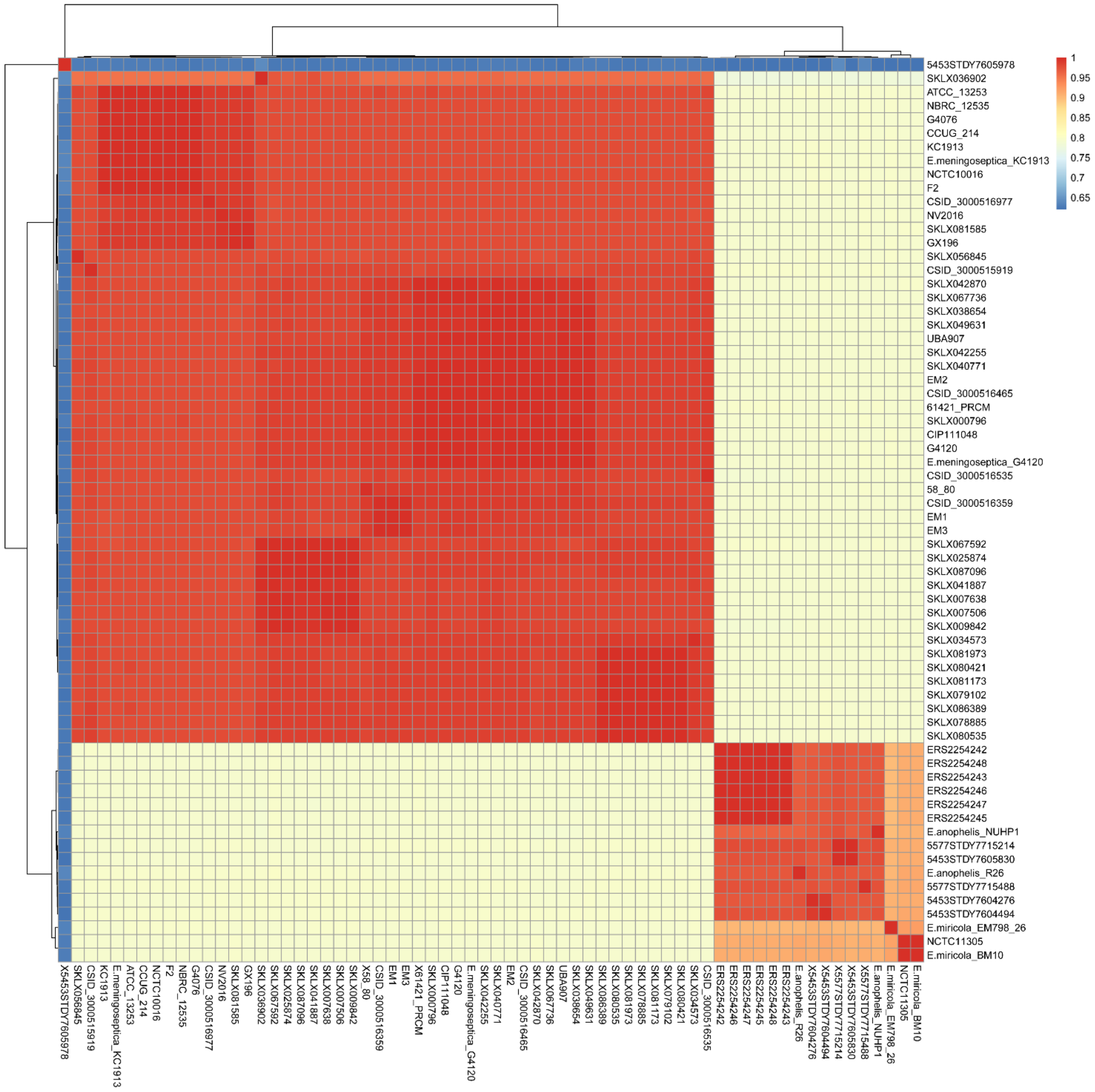


**Figure S7. The ANI value heatmap among all studied *Elizabethkingia* species.** A total of 64 strains are presented on the map, including 13 strains that have been incorrectly identified as *E. meningoseptica*. The ANI values of these 13 isolates are all < 80%.
